# Oxidative Stress Delays Prometaphase/Metaphase of the First Cleavage in Mouse Zygotes via the MAD2L1-Mediated Spindle Assembly Checkpoint

**DOI:** 10.1155/2017/2103190

**Published:** 2017-09-25

**Authors:** Que Wu, Zhiling Li, Yue Huang, Diting Qian, Man Chen, Wanfen Xiao, Bin Wang

**Affiliations:** Reproductive Center, The First Affiliated Hospital of Shantou University Medical College, Shantou University, Shantou, Guangdong, China

## Abstract

In zygotes, DNA damage delays the first cleavage to enable repair. Our previous study found that 0.03 mM hydrogen peroxide (H_2_O_2_) was the minimum concentration required for induction of oxidative DNA damage in mouse zygotes and that this represented the most similar situation to the clinical phenomenon. In this study, we quantified the cleavage rates of cells in blastocysts at different developmental stages, followed by immunofluorescence to detect activation of *γ*-H2A histone family member X (a marker of DNA damage) in zygotes to confirm that oxidative DNA damage was induced in H_2_O_2_-treated zygotes. Monitoring H3S10P (phosphorylation of Ser10 on histone H3; a prometaphase/metaphase marker) levels at different hour postinsemination revealed that treatment of zygotes with 0.03 mM H_2_O_2_ resulted in a prometaphase/metaphase delay. Furthermore, immunofluorescence staining for mitotic arrest deficient 2-like 1 and the protein kinase TTK, components of the spindle assembly checkpoint (SAC), suggested that this delay possibly involved SAC activation. These studies of the relationships between oxidative stress and SAC can promote the success rate of *in vitro* fertilization.

## 1. Introduction

Although many infertile couples have birthed children with the help of assisted reproduction technologies, these methods continue to suffer from excessive developmental failure due to differences between *in vitro* culture conditions and the *in vivo* environment, including energy sources, growth factors, pH, and atmosphere. Suboptimal embryo-culture conditions *in vitro* can induce high levels of reactive oxygen species (ROS) in embryos and alter embryo development [1–3]. Excessive ROS generation can affect cellular function, signal transduction, and gene expression, as well as lipid peroxidation and physical DNA damage, resulting in nuclear and mitochondrial DNA-strand breaks in embryos [1, 4, 5]. High levels of ROS represent a major contributing factor *in vitro* to developmental blockage in zygotes [6]. However, due to ethical limitations, research cannot be performed on human embryos; therefore, in our studies, we treat mouse zygotes with hydrogen peroxide (H_2_O_2_) to simulate the clinical phenomenon.

In the clinic, embryonic aneuploidy in gametes or early embryos represents the leading cause of *in vitro* fertilization (IVF) failure [7]. This occurs when abnormal chromosomes or their abnormal segregation events are present in early stage embryos [8], resulting in excessive developmental failure in embryos [9]. However, some embryos can appear normal by day 3, although many do not reach the blastocyst stage [10, 11]. This phenomenon might be related to ROS [12]; therefore, this study tested a hypothesis suggesting a possible relationship between ROS and aneuploidy occurrence in *in vitro-*cultured zygotes. The occurrence of aneuploidy is tightly connected with spindle assembly checkpoint (SAC) function, which ensures correct segregation of chromosomes into daughter cells and prevents premature metaphase-anaphase transition until all chromosomes successfully attach to the bipolar spindle with proper tension [13]. The SAC can prevent aneuploidy by delaying chromosome segregation at prometaphase or metaphase in response to unattached kinetochores and DNA damage during preimplantation development [9, 14–16].

Because the SAC is involved in monitoring the metaphase-anaphase transition, to determine whether the cell cycle checkpoint mediates metaphase delay in response to oxidative stress, we analyzed the cell cycle kinetics of the first cleavage event in the zygote by monitoring the number of 2-cell blastocysts with both nuclei enriched for histone H3 phosphorylated on Ser10 (H3S10P), a marker of prometaphase/metaphase [15, 17]. In mammalian cells, H3S10P initially occurs within a subset of genes in late G2-interphase cells but subsequently spreads along the chromosomes until completion at prometaphase, after which H3S10P is still evident at the metaphase plate [17, 18]. Delay of H3S10P events in whole chromatin is indicative of prometaphase/metaphase delay and SAC activation [15].

To determine whether the SAC is engaged in response to oxidative stress damage in zygotes, we examined the localization of mitotic arrest deficient 2-like 1 (MAD2L1; also known as MAD2) and the protein kinase TTK by immunofluorescence staining. The end of prometaphase in mitosis correlates with checkpoint inactivation and disappearance of MAD2L1, a key player involved in SAC function [19]. MAD2L1 is observed in a punctate pattern throughout the cytoplasm but translocates to the nucleus following DNA damage and SAC activation [15, 20]. By contrast, the TTK dual-specificity protein kinase, which has been implicated in regulation of mitotic progression, is encoded by human and mouse *monopolar spindle 1* (*MPS1*) [9] and is essential for the SAC. TTK can directly prevent anaphase onset in the presence of misaligned chromosomes. Additionally, TTK activity at the kinetochore stimulates MAD1-dependent conformational changes in MAD2L1 from an open to a closed form [21, 22], which is crucial for inhibition of anaphase-promoting complex/cyclosome (APC/C) [6, 23–26], the ubiquitin ligase that controls anaphase onset.

To characterize the effects of oxidative stress on zygotes, our previous studies used different doses of hydrogen peroxide (H_2_O_2_) to treat mouse zygotes at 7 h postinsemination (hpi) to simulate the clinical phenomenon of oxidative damage in zygotes. Our previous results indicated that 0.03 mM H_2_O_2_ was the minimum concentration capable of inducing higher intracellular ROS levels and triggering oxidative damage in the zygote [27, 28]. In this study, we treated zygotes with 0.03 mM H_2_O_2_ at 7 hpi (G1 phase) to induce oxidative stress in zygotes, finding that H_2_O_2_ reduced the rate of blastocyst formation at day 4 but did not reduce the rates of 2-cell formation, 4-cell formation, and 8-cell formation until day 3 as compared with that observed in untreated control zygotes, representing the most similar situation to clinically observed phenomena [29, 30]. Previous studies showed that embryo damage caused by suboptimal culture media during the early stages can reduce the percentage of surviving embryos reaching the blastocyst stage [10]. *γ*-H2A histone family member X (*γ*H2AX) plays an important role in DNA damage response [31] and is often used as a marker for DNA damage [32–34]. Therefore, to determine whether blastocyst reduction results from DNA damage, we identified whether DNA damage occurs in zygotes by immunocytochemical detection of *γ*H2AX protein.

Our previous study found that zygotes treated with 0.03 mM H_2_O_2_ display a delayed G2/M phase transition during the first cleavage and that the DNA damage response (DDR) pathway was involved in this delay [28]. However, we previously failed to distinguish whether the delay occurred at G2 phase or M phase in zygotes; therefore, in this study, we performed immunofluorescent identification of H3S10P, finding that treatment of zygotes with 0.03 mM H_2_O_2_ resulted in prometaphase/metaphase delay. Furthermore, immunofluorescence staining of MAD2L1 and TTK suggested that the SAC is involved in metaphase delay induced by oxidative stress.

## 2. Materials and Methods

### 2.1. Experimental Animals

Adult Kunming mice (3–6 weeks old) were obtained from the animal center of Shantou University Medical College. Treatment of all mice was in compliance with the Guide for the Care of Use of Laboratory Animals by the National Institutes of Health (NIH publication number 85–23, revised 1996) and the rules set forth by the National Animal Protection of China. All experimental protocols were approved by the Laboratory Animal Ethics Committee of our institution (SUMC2014-014). This study was approved by the Institutional Animal Care and Use Committee of Shantou University Medical College.

### 2.2. Reagents and Media

Rabbit antiphospho-histone H2AX (*γ*H2AX; Ser139), rabbit antiphospho-histone H3 (Ser10), and rabbit antiMAD2L1 were purchased from Abcam (Cambridge, UK). Rabbit anti-TTK was obtained from Proteintech (Chicago, IL, USA). Goat antirabbit secondary antibody was obtained from Santa Cruz Biotechnology (Dallas, TX, USA). Human tubal fluid (HTF) was obtained from Sage Science (Beverly, MA, USA). 4,6-diamidino-2-phenylindole (DAPI; 2 *μ*g/mL) was obtained from Sigma-Aldrich (St. Louis, MO, USA). Antifade fluorescence-mounting medium was purchased from Beyotime Institute of Biotechnology (Haimen, China).

Phosphate-buffered saline (PBS) was dissolved in 1 L ultrapure water: 8 g NaCl, 0.2 g KCl, 1.44 g Na_2_HPO_4_, and 0.24 g KH_2_PO_4_ (pH 7.4). Pancreatin solution was diluted to 0.1% with PBS, and the pH adjusted to 3.0 with HCl prior to and storage at 4°C. Zygote culture medium was prepared by adding 0.4% bovine serum albumin (BSA; Sigma-Aldrich) and 10% fetal bovine serum (FBS; Gibco; Thermo Fisher Scientific, Waltham, MA, USA). The medium for the treated group was created by adding H_2_O_2_ to a final concentration of 0.03 mM, followed by preequilibration for 1 h before use. Fixative liquid consisted of 4% paraformaldehyde in PBS, and permeabilization buffer consisted of PBS supplemented with 0.5% Triton X-100. Sperm capacitation liquid (HTF solution supplemented with 1.5% BSA), fertilization liquid (HTF solution supplemented with 0.4% BSA), and zygote culture medium were incubated at 37°C in a 5% CO_2_ incubator and allowed to equilibrate for 4 h before use.

### 2.3. Collection of Sperm and Oocytes, IVF, and Culture of Zygotes

As described in our previous studies [35, 36], male Kunming mice of reproductive age were euthanized, and sperm was collected from the murine cauda epididymis and incubated in capacitation medium (HTF medium containing 1.5% BSA) at 37°C in a 5% CO_2_ incubator for 1 h. Female mice were induced to superovulate by consecutive intraperitoneal injections of 10 IU pregnant mare serum gonadotropin and 10 IU human chorionic gonadotropin (HCG) 48 h apart [28], followed by euthanization at 13 h to 15 h after HCG administration to obtain cumulus oocytes from the oviducts. Cumulus oocytes were collected in 37°C PBS, followed by transfer to prepared 37°C fertilization liquid (HTF medium containing 0.4% BSA) under oil and containing 10 *μ*L capacitated sperm and incubation at 37°C for 6 h in a 5% CO_2_ incubator to permit fertilization. Zygotes were then washed three times and cultured in new medium (HTF medium supplemented with 0.4% BSA and 10% FBS).

### 2.4. Mouse Zygote Model for Oxidative Damage

According to our previous studies, 0.03 mM H_2_O_2_ is the minimum concentration capable of inducing oxidative damage in zygotes [27]. Therefore, at 7 hpi, we selected two pronuclear zygotes in G1 phase for exposure to culture medium containing 0.03 mM H_2_O_2_ for 30 min to induce oxidative damage. Zygotes in the control and treated groups were then extensively washed twice in fresh embryo-culture medium, followed by further culture in embryo-culture medium for evaluation.

### 2.5. Indirect Immunofluorescence Staining for *γ*H2AX, H3S10P, MAD2L1, and TTK

Zygotes were collected at different times to detect the activation of different proteins (*γ*H2AX was detected at 17.0-18.0 hpi, H3S10P was detected every 30 min at 18.0–24.0 hpi, and MAD2L1 and TTK were detected at 19.0–19.5 hpi and 21.5–22.5 hpi in the control and treated groups, resp.). Zona pellucida was removed from zygotes with 0.1% pancreatin. After washing for 5 min three times with PBS containing 0.05% Tween-20 (TPBS), the zygotes were fixed in 4% paraformaldehyde in TPBS for 30 min, mounted on polylysine-coated slides, and washed three times again with TPBS. Zygotes were then permeabilized in TPBS supplemented with 0.5% Triton X-100 for 30 min at room temperature. After washing three times as above, zygotes were blocked in blocking solution (TPBS supplemented with 10% normal goat serum and 3% BSA) for 1 h. After blocking, primary antibodies were incubated at 4°C overnight at the following dilutions: rabbit anti-*γ*H2AX (1 : 300), rabbit anti-H3S10P (1 : 150), rabbit anti-MAD2L1 (1 : 100), and rabbit anti-TTK (1 : 100). After washing with TPBS three times, zygotes were incubated with the secondary antibody (goat antirabbit IgG-FITC; 1 : 200) at room temperature for 1 h then washed three times with TPBS. Zygotes were counterstained with DAPI at room temperature for 30 min, washed three times, mounted with antifade fluorescence-mounting medium, and coverslipped. A fluorescence microscope (Nikon Eclipse90 Ni-E; Nikon, Tokyo, Japan) was used to observe the signal.

## 3. Results

### 3.1. Treatment with 0.03 mM H_2_O_2_ Reduces Blastocyst Formation without Affecting Early Cell Division

**Figure 1:**
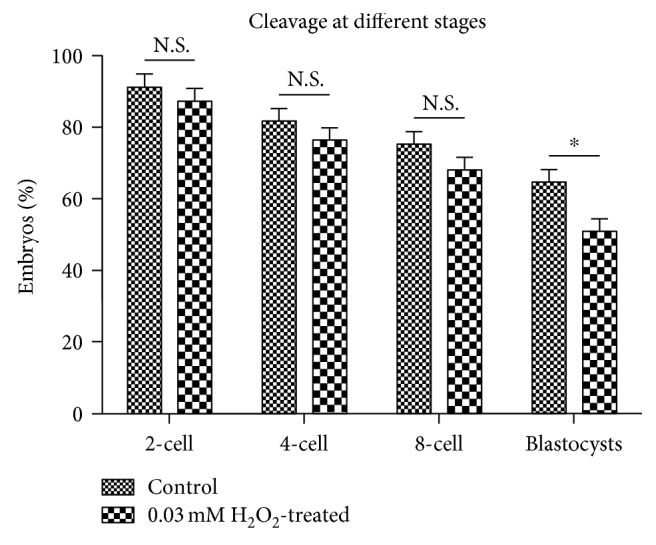
Developmental profiles of control and H_2_O_2_-treated embryos. Based on the chi-square test, no statistical differences were observed in the rates of 2-cell formation, 4-cell formation, or 8-cell formation between the control and treated groups (*P* > 0.05). However, treatment with 0.03 mM H_2_O_2_ produced a significant decrease in the rate of blastocyst formation (*P* < 0.05). ^∗^*P* < 0.05.

To explore the impact of oxidative damage on embryo development *in vitro*, we monitored cleavage rates at different stages in control and 0.03 mM H_2_O_2_-treated groups. Our results showed that H_2_O_2_ treatment did not significantly reduce the rates of 2-cell formation, 4-cell formation, or 8-cell formation (*P* > 0.05) but produced a significant decrease in the rates of blastocyst formation (*P* < 0.05) (Figure 1), which was similar to phenomena observed in the clinic [29, 30]. Therefore, we considered that treatment with 0.03 mM H_2_O_2_ reflected clinical relevance and was suitable for inducing oxidative damage in the treated group.

### 3.2. H_2_O_2_ Exposure Induces Nuclear *γ*H2AX Foci Formation in Mouse Zygotes

**Figure 2:**
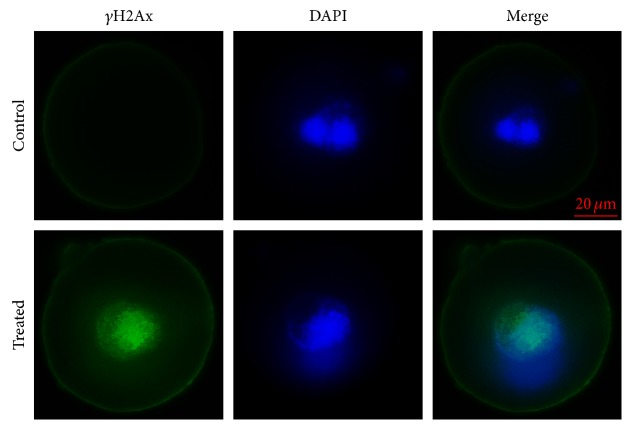
*γ*H2AX detection in mouse zygotes at 17 hpi. Nuclei were stained with DAPI (blue). *γ*H2AX staining was detected in the nuclei of H_2_O_2_-treated embryos, but not in those of the control group.

*γ*H2AX is an early and sensitive marker for DNA damage. To determine whether H_2_O_2_ induces DNA damage, we monitored the presence of *γ*H2AX staining by immunofluorescence microscopy. Punctate *γ*H2AX staining was observed in the nuclei of H_2_O_2_-treated zygotes but not detected in control zygotes (Figure 2). These findings indicated that H_2_O_2_ treatment resulted in DNA damage.

### 3.3. The Presence of H3S10P during Different Phases in Mouse Zygotes

**Figure 3:**
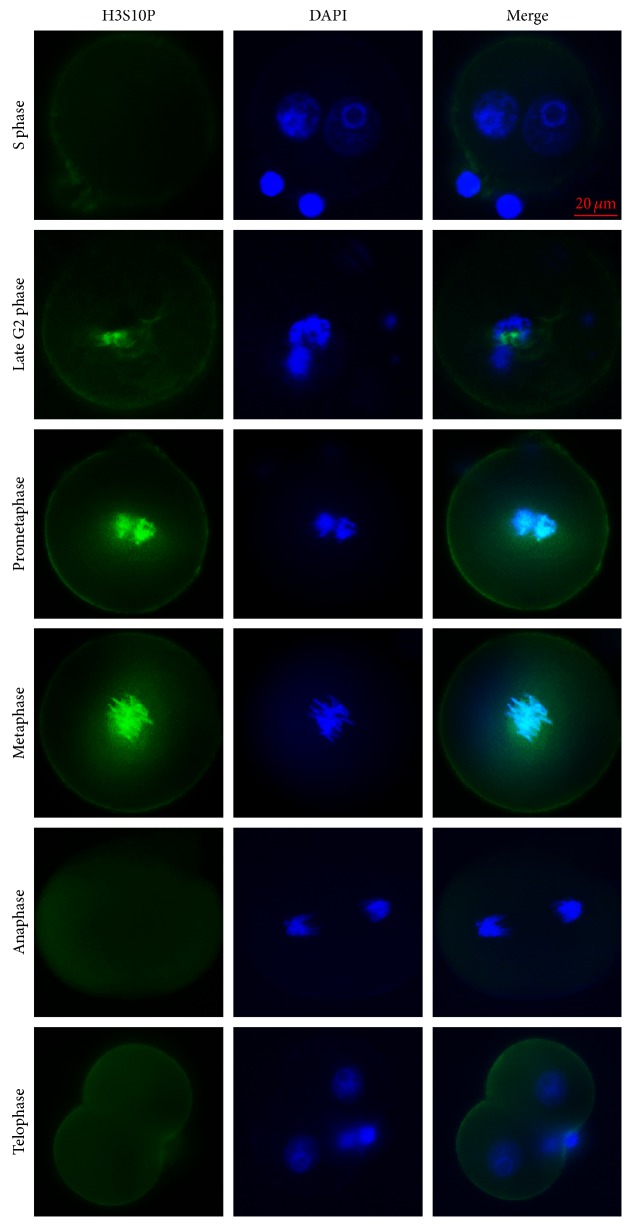
H3S10P detection in mouse zygotes. H3S10P (green) expression during S phase, prometaphase, and anaphase in control zygotes at 18.0, 19.0, and 20.5 hpi, respectively. The late G2 phase, metaphase, and telophase visualizations were obtained from treated zygotes at 21.0, 22.0, and 23.5 hpi, respectively. Nuclei were stained with DAPI (blue).

In our study, we observed no H3S10P-positive signals (green) during the S phase, and subsequent analysis indicated that H3S10P affected only a subset of genes and not the entire chromatin (blue staining with DAPI) in late G2-interphase cells. However, H3S10P prevalence extended along the chromosomes, with completion observed during prometaphase and remaining evident at the metaphase plate, but disappearing during anaphase and telophase (Figure 3). Therefore, we used H3S10P as a marker of prometaphase/metaphase during mitosis.

### 3.4. Percentage of H3S10P-Positive Zygotes at Different Time Postfertilization

**Figure 4:**
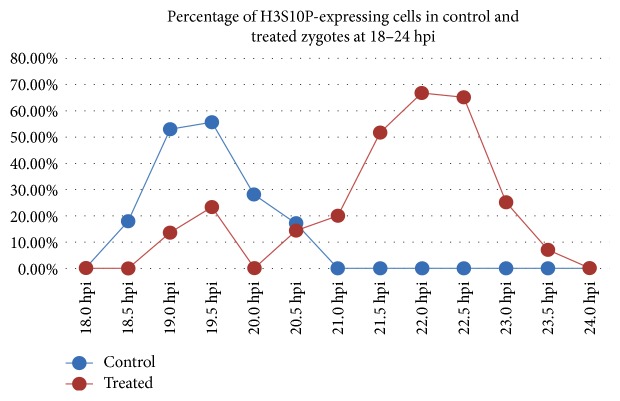
Percentage of cells showing H3S10P prevalence in control and treated zygotes from 18 hpi to 24 hpi. The peak of H3S10P prevalence occurred later in treated zygotes.

We monitored the ratios of prometaphase/metaphase zygotes by immunofluorescence staining for H3S10P at 30 min intervals and 18 hpi to 24 hpi (Table 1). After converting the ratios into percentages, we found that the percentage of H3S10P-positive zygotes in the control group was >50% at 19.0 hpi and 19.5 hpi (52.86% and 55.56%, resp.), reaching a maximum at 19.5 hpi and decreasing to zero at 21.0 hpi (i.e., when zygotes finished the first cleavage). However, in the H_2_O_2_-treated group, the percentage of H3S10P-positive zygotes was >50% at 21.5, 22.0, and 22.5 hpi (51.4%, 66.6%, and 65.0%, resp.) and reached a maximum at 22.0 hpi, after which H3S10P levels decreased rapidly and were no longer observed. These data suggested that prometaphase/metaphase was delayed in H_2_O_2_-treated zygotes and that the duration of prometaphase/metaphase in treated zygotes was prolonged relative to that observed in the control group based on the broader peak width observed in the H_2_O_2_-treated group (Figure 4).

### 3.5. MAD2L1 and TTK Expression in the H_2_O_2_-Treated and Control Groups

**Figure 5:**
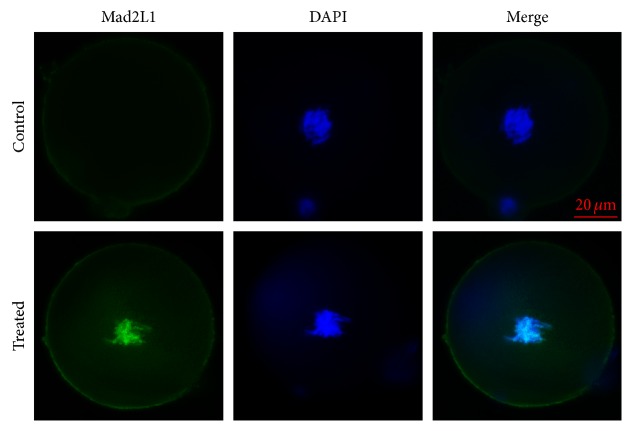
MAD2L1 expression is induced in mouse zygotes following treatment with 0.03 mM H_2_O_2_. MAD2L1 immunofluorescence (green) was undetected in untreated cells but detected following treatment of zygotes with H_2_O_2_. Nuclei (blue) were stained with DAPI.

**Figure 6:**
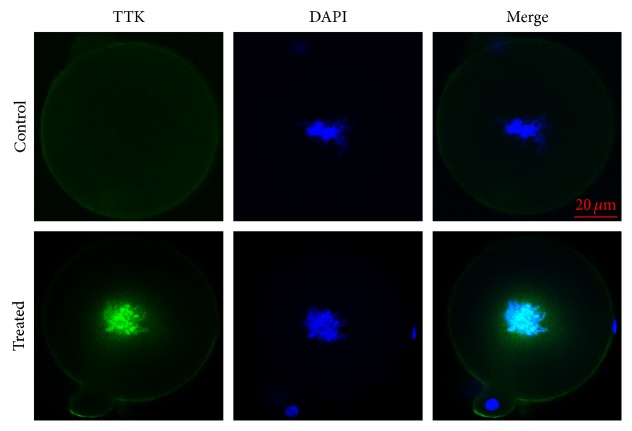
TTK expression in mouse zygotes is induced following exposure to 0.03 mM H_2_O_2_. TTK immunofluorescence (green) was undetected in untreated cells but detected following treatment of zygotes with H_2_O_2_. Nuclei (blue) were stained with DAPI.

Based on H3S10P prevalence, we chose to examine the expression of MAD2L1 and TTK from 19.0 hpi to 19.5 hpi for the control group and from 21.5 hpi to 22.5 hpi for the treated group. In control zygotes, MAD2L1 expression displayed a punctate pattern throughout the cytoplasm but was enriched in the nucleus in H_2_O_2_-treated zygotes (Figure 5, green). TTK expression was similarly analyzed by indirect immunofluorescence (Figure 6, green), showing positive staining in the H_2_O_2_-treated group in association with the chromatin and no staining detected in the control group. These results suggested that SAC was activated upon oxidative damage.

## 4. Discussion

Cultured embryos are usually subjected to oxidative stress *in vitro* due to suboptimal culture conditions, and H_2_O_2_ is commonly used in studies investigating the effects of oxidative stress [2, 27, 37]. When DNA damage results from oxidative stress, two pathways are activated in zygotes: the DDR pathway, which monitors DNA integrity, and the SAC, which responds to defects in spindle attachment/tension in metaphase during mitosis and meiosis [15]. We previously showed that oxidative damage activates the DDR pathway to mediate G2/M cell cycle arrest to allow repair of H_2_O_2_-induced oxidative damage [28]. By contrast, in cycling cells, the SAC is critical for preventing genome instability and producing healthy daughter cells containing the same genetic information as the mother cell during mitosis. In this study, we explored the timing of metaphase during the first round of mitosis in control and H_2_O_2_-treated zygotes and investigated the involvement of the SAC in metaphase delay.

*γ*H2AX is an early indicator of DNA damage [31], and we previously suggested that *γ*H2AX plays an important role in both DDR [28] and SAC by contributing to the recruitment of both TTK and MAD2L1 to the kinetochore during metaphase [38]. Our results in this study (Figure 2) showed that oxidative stress-induced DNA damage existed in H_2_O_2_-treated zygotes, but not in control zygotes. Additionally, we revealed that H_2_O_2_ treatment of zygotes did not reduce the rates of 2-cell formation, 4-cell formation, or 8-cell formation but did reduce the rates of blastocyst formation at day 4. This indicated that a moderate concentration of H_2_O_2_ (0.03 mM) produced a similar outcome as that observed clinically and in previous studies [27, 29, 30]. Therefore, this H_2_O_2_ concentration was used to treat embryos in our study.

H3S10P is a marker for prometaphase/metaphase [39] and can be used to identify metaphase delay upon DNA damage in zygotes [15]. Here, we showed that the peak percentage of H3S10P-positive cells in untreated embryos occurred at 19.5 hpi and that after 21 hpi, H3S10P was no longer detected as a result of all control zygotes having finished their cleavage. However, in the H_2_O_2_-treated group, the percentage of H3S10P-positive cells peaked at 22.0 hpi, indicating that prometaphase/metaphase entry was delayed. Additionally, the duration of prometaphase/metaphase in treated zygotes was prolonged relative to that observed in the control group. Because the SAC can delay chromosome segregation at prometaphase or metaphase in response to DNA damage [14, 15], we considered the delay observed in the treated group as resulting from SAC activation. Furthermore, we observed a small peak at 19.5 hpi in the treated group prior to the appearance of the maximum peak and suggesting zygote heterogeneity in response to oxidative damage, with some zygotes either being insensitive to mild oxidative damage or exhibiting an inactive checkpoint. Based on these results, we chose times from 19.0 hpi to 19.5 hpi and 21.5 hpi to 22.5 hpi for detection of the SAC markers MAD2LI and TTK in control and treated zygotes, respectively.

The metaphase-anaphase transition is mediated by the ubiquitin protein ligase APC/C [40] under the control of a network of regulatory factors, including cell division cycle protein 20 (CDC20), cadherin 1 (CDH1), and MAD2L1. CDC20 and CDH1 can activate APC, whereas MAD2L1 acts as an APC/C inhibitor. When the SAC is activated, MAD2L1 forms a ternary complex with CDC20 and the protein kinase BUBR1 to form the mitotic checkpoint complex (MCC) and prevent APC/C activation, thereby arresting cells at prometaphase [41]. As a critical mediator of MCC formation and APC/C activation [26, 42], MAD2L1 also plays a key role in SAC functions [19]; therefore, its detection represents a significant determinant of SAC activated. Here, our results (Figure 5) showed that MAD2L1 was enriched in the nucleus in response to oxidative damage, providing further evidence of SAC activation following oxidative damage in zygotes. The SAC monitors microtubule tension and attachment at kinetochores to ensure proper chromosome segregation [43], and MAD2L1 localization in the nuclei is regulated by attachment, not tension. Even a single unattached kinetochore is sufficient to activate MAD2L1 nucleocytoplasmic transport and delay anaphase [20]. Therefore, our findings suggested that oxidative stress was capable of activating the SAC based on dysregulated kinetochore-microtubule attachment.

In addition to the spatial and temporal regulation of MAD2L1 localization during cell cycle progression and SAC activation, two MAD2L1 conformations with distinct topologies are also vital to the SAC: the open (O) and closed (C) MAD2L1 conformation [26]. Only the C-MAD2L1 conformation obtained following associating with MAD1 or CDC20 can allow blockage of anaphase progression by inhibiting APC/C activation for SAC signaling [22, 26, 42]. A significant function associated with TTK involving cell cycle control concerns conversion of cytosolic MAD2L1 from an inactive open conformation (O-MAD2L1) to a closed conformation (C-MAD2L1) [21]. Another important finding of this study was elevated TTK expression in zygotes exposed to oxidative stress, suggesting a relationship between TTK expression and oxidative stress. Our findings also suggested that during SAC signaling, MAD2L1 recruitment to kinetochores by TTK also occurred upon the initiation of oxidative damage in zygotes [21, 22, 27]. However, TTK only appears to be required for SAC function following spindle damage and not during unperturbed mitosis [44, 45]. By contrast, other SAC components restrain mitosis in the absence of spindle damage [45]. Therefore, as an indicator of SAC activation, our observation of TTK expression in H_2_O_2_-treated zygotes represented additional evidence of SAC activation following oxidative damage.

The SAC is required for regulating mitotic cell cycle progression to ensure mitotic fidelity during preimplantation development in mouse cleavage-stage embryos [9, 46]. The primary role of the SAC is to ensure that all chromosomes are bioriented and accurately attached to spindle microtubules prior to anaphase initiation, thereby contributing to the prevention of aneuploidy by blocking anaphase onset until correct kinetochore-microtubule attachment and tension are attained [16]. Otherwise, errors in chromosome segregation or distribution might result in aneuploid-embryo formation, potentially resulting in implantation failure, spontaneous abortion, genetic diseases, or embryonic death [9]. Embryonic aneuploidy in gametes or early embryos is already the leading cause of IVF failure [7, 9]. Studies of the relationships between oxidative stress and SAC aid in promoting strategies to avoid spontaneous abortion and low rates of reproductive success. However, a more detailed understanding of the mechanisms associated with SAC activation and aneuploid-embryo formation related to oxidative stress is necessary.

## 5. Conclusion

Our results showed that treatment of zygotes with 0.03 mM H_2_O_2_ did not influence the formation of 2-cell embryos, 4-cell embryos, or 8-cell embryos but did reduce rates of blastocyst formation, which was similar to developmental phenomena observed clinically in relation to embryos experiencing oxidative damage. Additionally, we observed DNA damage H_2_O_2_-treated zygotes based on the detection of *γ*H2AX, the induction of metaphase delay during initial mitosis of zygotes, and the time of appearance of H3S10P. Furthermore, detection of MAD2L1 and TTK expression by immunofluorescence suggested the involvement of SAC activation in this delay. Given that SAC is largely responsible for chromosome stability, its activation is conducive to avoiding spontaneous abortion resulting from aneuploidy.

## Figures and Tables

**Table 1:** Ratio of H3S10P-expressing cells in control and treated zygotes from 18 hpi to 21 hpi.

Groups	18.0 (hpi)	18.5 (hpi)	19.0 (hpi)	19.5 (hpi)	20.0 (hpi)	20.5 (hpi)	21.0 (hpi)	21.5 (hpi)	22.0 (hpi)	22.5 (hpi)	23.0 (hpi)	23.5 (hpi)	24.0 (hpi)
Control	0/67	10/56	37/70	40/72	21/75	12/70	0/63	0	0	0	0	0	0
Treated	0/52	0/53	7/52	20/86	0/49	10/70	12/60	36/70	46/69	39/60	16/64	4/58	0

The treated group was administered 0.03 mM H_2_O_2_. Hpi: hours postinsemination.
